# Effect of Skin-To-Skin Contact on Preterm Infant Skin Barrier Function and Hospital-Acquired Infection

**DOI:** 10.4021/jocmr479w

**Published:** 2011-02-12

**Authors:** Amel Abouelfettoh, Susan M. Ludington-Hoe, Chris J. Burant, Marty O. Visscher

**Affiliations:** a Case Western Reserve University Bolton School of Nursing, USA; b Cairo University Faculty of Nursing, Egypt; c Skin Sciences Institute, Cincinnati Children's Hospital Research Foundation, USA

## Abstract

**Background:**

The preterm infants' skin is structurally and functionally immature at birth because of immature stratum corneum barrier function, leading to problems with fluid loses, thermoregulation, and infection. Two parameters of barrier function can be non-invasively assessed: Stratum Corneum Hydration (SCH) and Transepidermal Water Loss (TEWL). Skin-to-Skin Care (SSC) is the proposed independent variable that might affect barrier function by decreasing TEWL and increasing SCH, thereby improving stratum corneum barrier function and consequently decreasing the rate of infection. No study of SSC's effects on TEWL and SCH of preterm infants could be found. The purpose of the study was to determine the effect of 5 daily Skin-to-Skin Contact sessions on infant skin hydration (SCH), transepidermal evaporated water loss (TEWL), and on SCH when TEWL was controlled, and on the presence of hospital acquired infection.

**Methods:**

A one-group pretest-test-posttest design with 10 preterm infants (28 - 30 wks GA < 32 wks postmenstrual age, and no infection at entry). Test = 90 minutes of SSC; pre-test and post-test = 30 minutes each of prone positioning in an incubator. SCH and TEWL were taken on Days 1 and 5 at the beginning, middle and end of each period using Multi-Probe Adaptor. A 3 X 3 X 2 Repeated Measures Mixed Models Design, including a covariate, was used to analyze level of Skin Hydration. Specifically, the model tested comparisons in SCH made across repetitions, time, and days, as well as all possible interactions while controlling for TEWL. Descriptive statistics described the number of positive blood cultures during hospitalization and the presence of infections four weeks post-discharge.

**Results:**

Significant differences in skin hydration were found across TIME (Pre-SSC, SSC, Post-SSC) (F = 21.86; p < 0.001). One infant had a positive blood culture during hospitalization; no infants had signs of infection by 4 weeks post-discharge.

**Conclusions:**

The study has begun fulfilling the recommendation that SSC be tested as a strategy to improve skin hydration, but reveals that evaporative loss may be higher during SSC than during incubator care, and that the higher transepidermal evaporated water loss values may not necessarily be detrimental because few infections occurred even in its presence. A definitive randomized controlled trial is recommended.

**Keywords:**

Skin-to-skin contact; Skin hydration; Transepidermal water loss; Infection; Preterm

## Introduction

Because the skin is a common point of entry for invasive pathogens, interventions to improve the preterm infant's skin barrier functions are needed [1]. The skin of premature infants has the functions of inhibiting water loss (called transepidermal evaporated water loss (TEWL)) from the tissues, and maintaining adequate moisture (stratum corneum hydration (SCH)) through the stratum corneum (SC) to prevent entry of damaging microorganisms. But, skin barrier functions are compromised due to immaturity [2]. The premature infant's immature skin lacks vernix casseosa, a protective cutaneous biofilm with antimicrobial properties [3]. When prematurity becomes more extreme, the skin barrier is increasingly immature, the skin is more easily damaged and more functionally compromised [4]. The SC layer is also thinner and less well-developed than in full-term infants and adults, leading to high water loss, cutaneous infections, and high morbidity and mortality [5]. Consequently, hospitalized preterm infants suffer from the risks of infection and infection's co-morbidities [6-10]. Infection per se in preterm infants is also associated with increased risk of death [1] and increased medical cost [11-13].

Successful interventions to reduce infections, especially hospital acquired infections, would improve survival, reduce neonatal morbidity, and reduce the high medical and social costs of preterm infant care [5, 14-16]. Many interventions to reduce infection have been tested and are in use, such as hand-washing, administration of antibiotics and immunoglobulins, and isolation of the infant [2, 15, 17-20], but infections continue to occur. However, preterm infants experiencing skin-to-skin contact (SSC) have had fewer infections than infants who did not receive SSC [21, 22].

Skin-to-skin contact is skin-to-skin, chest-to-chest placement of the infant with mother at an incline of 30 - 40 degrees. Skin-to-skin contact may help reduce infections by improving the preterm infant's skin barrier function. A decrease in TEWL and an increase in SCH along all areas of infant skin that are in direct contact with maternal skin could occur and would be indicators of improved skin barrier function. Intermittent exposure to a high-humidity environment during feeding has been shown to quicken maturation of the skin barrier [23], leading the authors to hypothesize that intermittent exposure to the high-humidity microenvironment of SSC may do the same. No study of SSC's effects on TEWL and SCH of preterm infants could be found. Thus, the purposes of the study were to test for differences in TEWL and SCH between incubator (control condition) and SSC (experimental condition) on the first and last day of five daily SSC sessions within the same subjects, with each subject acting as his own control, and to determine the effect on SCH when TEWL was statistically controlled because SCH is a function of TEWL. The hypotheses were that 1) TEWL would be lower and SCH would be higher in SSC than in the incubator on Days 1 and 5, and 2) differences on Day 5 would be more favorable (lower TEWL and higher SCH) than differences on Day 1. One research question was posed: (because improved SCH and TEWL would enhance skin barrier function) what was the number of hospital-acquired infections (number of positive blood cultures during hospitalization and maternal report of any signs of infection at four weeks post-discharge) in infants who received SSC?

### Theoretical framework (relationship between SSC and skin barrier function)

Skin-to-skin contact may enhance SC barrier function in two ways. The first way is that TEWL may be minimized due to decreased skin exposure to evaporative heat loss during SSC [24, 25] or due to the "heat sink" microenvironment encountered when the infant lies contained between maternal breasts [26]. The environment between maternal breasts is a "heat sink" because the humidity and temperature are higher between the breasts than they are in an incubator, thereby preventing evaporative losses across the skin [27]. The second way in which SC barrier function may be enhanced is that the higher humidity microenvironment of SSC may reduce TEWL [28], resulting in a subsequent increase in skin hydration, a finding suggested by reports that mothers have commented about "sweating" during SSC, so that SSC is expected to provide the infant with more hydration resulting from mother's sweats [26, 29].

## Methods

### Design

A one-group, preSS-SSC-postSSC quasi-experimental one group design was conducted in which each subject served as his/her own control. Premature infants were tested during one inter-feeding interval on Days 1 and 5 of a five-day study in which infants were given SSC each day for five consecutive days. The inter-feeding interval was divided into three periods: a 30-minute pre-SSC period in the incubator, followed by a 90-minute SSC period, and then another 30-minute post-SSC period in the incubator immediately after SSC ended. On Days 1 and 5, TEWL and SCH were assessed three times in each period: at 10, 20, and 30 minutes of the pre-SSC and post-SSC periods, and at 30, 60, and 90 minutes during the SSC period, so that early, middle, and end assessment data for each period were available. On Days 2, 3, and 4, SSC was given for at least 30 and no more than 120 minutes each day. Signed informed consent was obtained following institutional review board approval at a university hospital in the Midwest. One research associate administered all assessments and monitored computerized data acquisition.

### Setting

Preterm infants were tested in a tertiary university-based Neonatal Intensive Care Unit which consisted of 7 rooms, each room housing 3 - 5 preterm infants of varying severity of illness who were separated by screens. Ambient temperature and humidity were recorded immediately before each TEWL and SCH reading during the pre-SSC, SSC, and post-SSC periods.

### Sample

Twenty-five mother-infant pairs were eligible over a 12 months period and 17 gave consent. Seven of the consented pairs did not begin data collection because of infant illness (n = 3) or withdrawal at maternal request due to hardship in providing 5 consecutive days of SSC (n = 4). Convenience sampling resulted in 10 mother-infant pairs who provided complete data sets.

### Subjects

Preterm infants born between 28 - 30 weeks gestation who met the following criteria were recruited: 1) were 30 - 31 weeks postmenstrual age at time of entry, so one week of study could be completed prior to the infant being 33 weeks post-conception age, the age at which the stratum corneum is usually as mature as the term infant's skin [23, 30], 2) were no more than 2 and 2/7 weeks old because the stratum corneum matures by completion of the third week of life [4] in infants living in an environment with a relative humidity less than 85% [23] which was the condition for all infants in the study, 3) were not receiving oxygen support, so transfer into and out of SSC was not complicated by oxygen lines, and 4) were not having central lines at time of study because central lines could be a source of infection as suggested by Asembergiene and colleagues [31]. Mothers were at least 18 years of age so they could give consent, were able to speak English so they could give informed consent, were willing and able to come for 5 consecutive days for SSC, and had no signs of skin rash or upper respiratory infection at time of entry. Infant exclusion criteria were 1) a previous infection any time since birth because previous infection indicates high susceptibility to re-infection [31], and 2) an impending (as determined by an increase in apnea, bradycardia and desaturation episodes, poor feeding, irritability, or lethargy) or active infectious process (as determined by positive blood cultures and/or administration of antibiotics and/or a diagnosis to rule out infection).

### Conditions

Feedings for all subjects were scheduled every three hours. For preSSC and postSSC periods the infants remained in double-walled incubators, and were positioned flat, prone, and nested while wearing only a diaper and a head cap. Transfer into SSC was accomplished by sitting transfer and infants were placed skin-to-skin, chest-to-chest between the breasts so that the infant's head was above the level of the mother's nipples and the head was turned to one side and erect (not flexed). During SSC infants wore diapers and had a receiving blanket folded-in-fourth covering their backs. The mother's hospital gown was closed over the infant's back. Mothers reclined at a comfortable level in a stationary zero-gravity lounger (La Fuma, France) behind privacy screens. Between Days 1 and 5 mothers agreed to provide daily sessions of SSC that were at least 65 minutes long.

### Outcome measures

SCH was defined as the amount of water retained by stratum corneum cells in the skin. Stratum corneum hydration is measured by changes in skin surface electrical capacitance and determined by the Moisture Accumulation Test [32]. SCH was measured as the moisture accumulation using the Corneometer® CM 825 [33]. The Corneometer probe was placed vertically against the infant's skin without pressure at a mark two centimeters below the infant's left nipple at the midclavicular line. The probe was held in constant contact for six seconds; SCH was recorded every second. The Corneometer-probe started the SCH measurement as soon as it came in contact with skin. A beep signaled that the SCH measurement had been successfully obtained, and once six measurements were obtained the probe was removed.

TEWL was defined as the rate of water evaporation from the skin and reported as g/m^2^/hr. Because TEWL is reported as a rate of evaporation rather than an amount of evaporation, TEWL was computed by averaging the data recorded every one second for 20 - 120 seconds determined by the shortest time to achieve 5 consecutive readings with a standard deviation < 0.1. The TEWL was measured by a Tewameter® TM 300 [33]. The TEWL probe was warmed up for 2 minutes before the first TEWL measurement was taken, and then the probe was turned on and manually held constantly against the infant's skin without pressure two centimeters below the infant's left nipple at the midclavicular line. The probe was maintained on the infant's skin for at least 20 seconds and no more than 120 seconds, detecting one measurement every second until the standard deviation between 5 consecutive readings was < 0.1, at which time the device automatically terminated readings [33].

Number of infections was defined as number of positive blood cultures since the day of entry into the study to the day of infant discharge from the hospital. Positive blood cultures were determined by examining daily lab reports.

Signs and symptoms of infection after discharge were defined as a positive maternal response to any one or more of the following questions: Has your infant had any fever since leaving the neonatal intensive care unit? Has your infant been diagnosed as having an infection since leaving the neonatal intensive care unit? Has your infant received any antibiotics during the first thirty days after leaving the neonatal intensive care unit?

### Instruments

To measure TEWL and SCH, a Multi-probe Adapter Systems MPA® [33] were used. The MPA system accommodated two probes: one probe was the Corneometer® CM 825 probe to measure SCH. The other probe was the Tewameter® TM 300 probe to measure TEWL. The MPA system was connected to a laptop computer with MPA software. SCH and TEWL measures were continuously downloaded from the MPA to the computer in real time in Excel format. The Tewameter and Corneometer probes did not need recalibration with each use, but calibration of the probes was checked once every month. The accuracy of the Tewameter probe was ± 0.5 g/hm for Relative Humidity ≥ 30% and ± 1.0 g/hm for Relative Humidity ≤ 30%. The accuracy of the Corneometer probe was ± 3.0% at optimal Relative Humidity of 30% - 70%. To ensure accurate function of the MPA probes, the MPA system was stored under the recommended storage conditions (Temperature: 0 70 C, Relative Humidity: 0% - 80%). Probes were warmed up for two minutes before measurements were taken and were not removed from the neonatal intensive care unit once the study began.

A Fisher Scientific Traceable Thermistor/Clock/Humidity Monitor (Model 06-662-4) was used to measure ambient temperature and relative ambient humidity. The monitor's accuracy was ± 1 ^o^C for ambient temperature, and ± 4% for relative humidity, and was calibrated biennially by the manufacturer.

Ohmeda Care Plus incubator skin probe was used to obtain infant skin temperature. The probe was placed one centimeter below the right costal margin and covered by a Mylar temperature shield (Accutemp Plus, Hayward, CA) to minimize ambient air and light influences. The probe is a 1.5 centimeter diameter flat metal disk that was attached to the abdomen one centimeter below the right costal margin at the mid-clavicle line.

### Procedure

Following signed consent, mothers and the research associate mutually agreed to a start date for SSC (Day 1). All measurements were taken on Days 1 and 5. The infant was positioned prone and left undisturbed in the incubator for the pre-SSC period. Ambient temperature and relative humidity were recorded at the beginning of each period. Assessments of infant skin temperature, TEWL, and SCH were recorded at Minutes 10, 20, and 30 of the pre-SSC and post-SSC periods. The SSC period began as soon as infant was transferred to mother's chest. Infant abdominal skin temperature, SCH, and TEWL assessments were recorded at Minutes 30, 60, and 90 of the SSC period. At the end of SSC, the infant was transferred back to the incubator and positioned the same as in the pre-SSC period. Infants were undisturbed by staff between measurements. On Days 2, 3, and 4, infants received SSC for 65 - 120 minutes, beginning whenever the mother came to visit. SSC was given only once each day.

### Data analysis

Subjects' demographics, TEWL, SCH, number of positive blood cultures during hospitalization, and the presence of signs of infection four weeks post-discharge were described using measures of central tendency and dispersion (Mean and SD). In the current study, data were collected from 10 subjects over 18 time points. The main focus was comparing Skin Hydration across three TIME periods (Pre-SSC, SSC, and Post-SSC). In order to take advantage of collecting data at 18 time points, a Repeated Measures Mixed Model approach was used to analyze the data, an approach that provided more power to analyze the data because the effective sample became 180 data points (10 subjects X 18 data collection points) as compared to 10 data points based on subjects alone. Data were collected in three ASSESSMENTS of data collection points for each of the three TIME periods across two DAYS. With this in mind, the final model was based on a 3 X 3 X 2 Repeated Measures Mixed Model design. This model allowed for the testing of the three ASSESSMENTS of data collection points across three PERIODS across two DAYS. Typically, in using a Repeated Measures Mixed Models Approach, an error covariance structure has to be identified for the repeated time components of the model. In the Current Model, a Compound Symmetry error covariance structure was applied to the PERIOD component. Testing of the ASSESSMENTS component indicated that an error covariance structure did not need to be applied as ASSESSMENTS demonstrated an independent error structure. Additionally, TEWL was included as a covariate, because TEWL impacts levels of Stratum Corneum Hydration. With this model, comparisons in SCH can be made across ASSESSMENTS, PERIODS, and DAYS, as well as the testing of interactions comparing ASSESSMENTS across PERIODS, ASSESSMENTS across DAYS, PERIODS across DAYS, and ASSESSMENTS across PERIODS across DAYS. TEWL was also analyzed using the same 3 X 3 X 2 Repeated Measures Mixed Model design. As with the previous model, a Compound Symmetry error covariance structure was applied to the TIME component, while the ASSESSMENTS component had an independent error structure.

## Results

Infant and maternal characteristics are presented in Table 1. Of note was the racial distribution of the subjects because 9 of the 10 subjects were Black. The total amount of SSC received daily on Days 2 - 4 was a mean of 77.5 (SD = 12.5) minutes. Ambient temperatures and infant skin temperature were not significantly different across all periods on Days 1 and 5. However, ambient humidity, as expected, was higher in the incubators than in the rooms during SSC (p = 0.001) (Table 2).

**Table 1:** Mothers and Infants Demographic Characteristics (N = 10)

Characteristics	Mean ± SD	N
Mothers Age	24.6 ± 4.03	
Infants GA	28.6 ± 1.26	
Infants Age at Entry	15.2 ± 7.19	
Minutes of KC/day (Day 2 - 4)	77.5 ± 12.5	
Infants Birth Weight	1228.4 ± 167.6	
Infants Weight at day one	1183.7 ± 331.8	
Infants Weight at day five	1348 ± 173.9	
Male/Female		8/2
AA/Caucasian		9/1
Vaginal/Cesarean Delivery		4/6
Breastfeeding/No Breastfeeding		3/7

**Table 2:** Means ± SDs for Ambient Humidity and Temperature and Infant Skin Temperature During Study Periods on Days 1 and 5

	Day 1			Day 5		
	Pre-SSC	SSC	Post-SSC	Pre-SSC	SSC	Post-SSC
Ambient Humidity	44.9 ± 12.2	27.3 ± 7.1	38.6 ± 12.7	39.1 ± 11.7	28.8 ± 7.3	36.1 ± 9.0
Ambient Temperature	30.7 ± 0.7	27.2 ± 0.7	30.3 ± 0.9	30.2 ± 1.9	27.1 ± 1.3	29.9 ± 2.1
Infant Skin Temperature	36.7 ± 0.3	36.8 ± 0.2	36.7 ± 0.3	36.7 ± 0.4	37.0 ± 0.2	36.6 ± 0.2

* p < 0.001 across all periods during both days

### SCH

**Figure 1.**
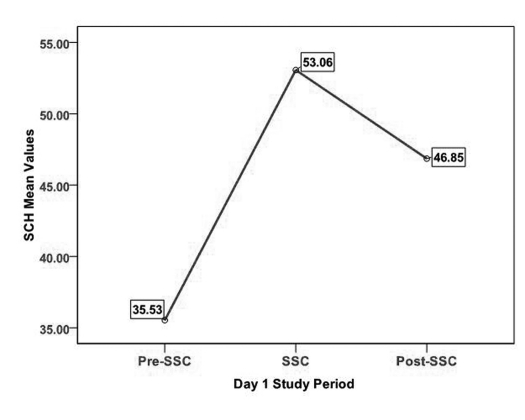
SCH on Day 1 across study periods.

**Figure 2.**
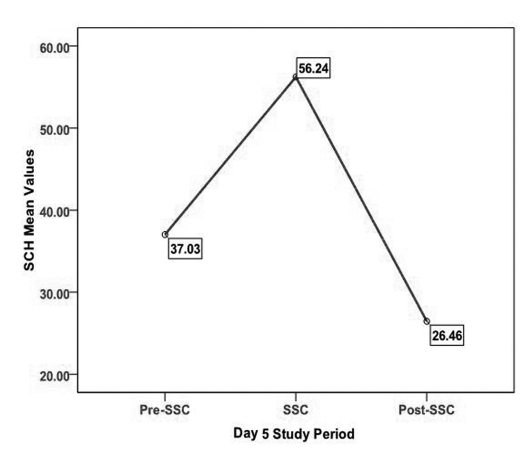
SCH on Day 5 across all study periods.

SCH mean values during each period on Days 1 and 5 are presented in Table 3. SCH demonstrated a pattern of increase from pre-SSC to SSC and then a decrease from SSC to post-SSC on Days 1 and 5 (Fig. 1, 2). A 3 X 3 X 2 Repeated Measures Mixed Models Design including a covariate was used to analyze level of SCH. Specifically, the model tested comparisons in SCH made across ASSESSMENTS, PERIODS, and DAYS, as well as all possible interactions while controlling for TEWL. In examining the main effects differences in ASSESSMENTS in data collection points and DAYS of SCH were not significant. However, significant differences in SCH were found across PERIODS (Pre-SSC, SSC, Post-SSC) (F (2, 18) = 21.86; *p* < 0.001). It should be noted that all mean scores are based on Least Squares Mean estimates. In examining the differences in SCH across PERIODS, SCH scores during the Pre-SSC period (LSMeans = 37.41) were significantly lower than SCH scores during the SSC period (LSMeans = 53.87) (Tukey-Kramer adjusted *p* < 0.001). Stratum corneum hydration scores during the Pre-SSC period (LSMeans = 37.41) were significantly lower than SCH scores during the Post-SSC period (LSMeans = 48.68) (Tukey-Kramer adjusted *p* < 0.001). However, only a trend was found comparing SCH scores during the SSC period (LSMeans = 53.37) and SCH scores during the Post-SSC period (LSMeans = 48.68) (Tukey-Kramer adjusted p = 0.09). All of these scores were adjusted for TEWL, however a non-significant trend was found for TEWL's influence on SCH (F (1,152) = 3.72; *p* = 0.06).

**Table 3:** SCH Means and SD Across Periods for Day 1 and Day 5 of the Study

	Pre-SSC	SSC	Post-SSC
	Mean ± SD	Mean ± SD	Mean ± SD
Day one	35.53 ± 14.69	53.06 ± 15.10	46.85 ± 12.67
Day five	37.02 ± 7.56	56.24 ± 17.84	51.22 ± 13.86

With regard to interaction effects, the only significant interaction effect was based on comparisons in SCH across ASSESSMENTS in data collection points and PERIODS (Pre-SSC, SSC, Post-SSC) (F (4, 36) = 2.83; *p* < 0.05). Among the 36 comparisons based on interactions, 10 significant differences were identified. Consistently, SCH at the second and third assessments during SSC and at the first assessment of Post-SSC were significantly larger as compared across assessments during Pre-SSC (Table 4). The first SCH assessment during SSC was not significantly different from any of the three assessments in Pre-SSC. As previously mentioned, the second SCH assessment during SSC was significantly larger than the first (Tukey-Kramer adjusted *p* < 0.05), the second (Tukey-Kramer adjusted *p* < 0.01), and the third (Tukey-Kramer adjusted *p* < 0.01) assessments during Pre-SSC. The third SCH assessment during SSC was significantly larger than the first (Tukey-Kramer adjusted *p* < 0.01), the second (Tukey-Kramer adjusted *p* < 0.001), and the third (Tukey-Kramer adjusted *p* < 0.001) assessments during Pre-SSC. A similar trend in comparing the first SCH assessment during Post-SSC and the three Pre-SSC assessments was found. Specifically, the first Post-SSC assessment was significantly larger than the first (Tukey-Kramer adjusted *p* < 0.05), the second (Tukey-Kramer adjusted *p* < 0.01), and the third (Tukey-Kramer adjusted *p* < 0.01) assessments during Pre-SSC. Additionally, the third SCH assessment during SSC was significantly larger than the third assessment of Post-SSC (Tukey-Kramer adjusted *p* < 0.05). These findings reflect that SSC, as compared to Pre-SSC, increased levels of SCH not significantly at the first assessment, but significantly through the second and third assessments during SSC and carried over onto the first assessment of Post-SSC. Interestingly, this carry over did not continue onto the third assessment of Post-SSC, where SCH levels were significantly lower as compared to the third assessment during SSC.

**Table 4:** Least Squares Means of SCH - Pair Wise Comparisons of PreSSC Period by SSC Period and PostSSC Period (Based on the Interaction Between Assessments and Periods)

		Pre-SSC Assessment 1	Pre-SSC Assessment 2	Pre-SSC Assessment 3
		LS Mean = 38.42	LS Mean = 36.55	LS Mean = 37.26
**SSC Assessment 1**	LS Mean = 49.56	Ns	Ns	Ns
**SSC Assessment 2**	LS Mean = 54.75	*P* < 0.05	*P* < 0.01	*P* < 0.01
**SSC Assessment 3**	LS Mean = 57.30	*P* < 0.01	*P* < 0.001	*P* < 0.001
**Post-SSC Assessment 1**	LS Mean = 53.82	*P* < 0.05	*P* < 0.01	*P* < 0.01
**Post-SSC Assessment 2**	LS Mean = 49.52	Ns	Ns	Ns
**Post-SSC Assessment 3**	LS Mean = 42.72	Ns	Ns	Ns

### TEWL

**Figure 3.**
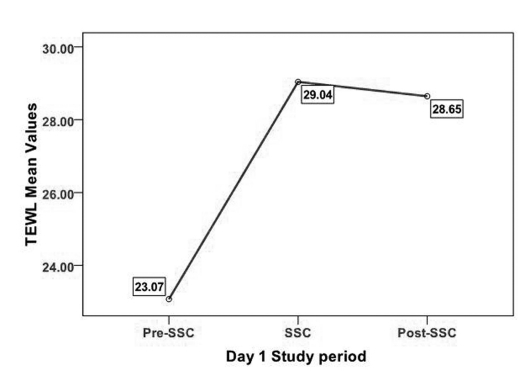
TEWL on Day 1 across all periods.

**Figure 4.**
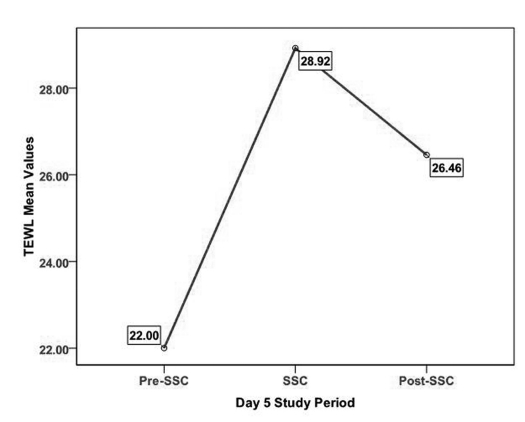
TEWL on Day 5 across all periods.

TEWL demonstrated a similar pattern of increase from pre-SSC to SSC and then decrease from SSC to post-SSC on Days 1 and 5 (Table 5 and Fig. 3, 4). A 3 X 3 X 2 Repeated Measures Mixed Models Design was used to analyze of TEWL across time. Specifically, the model tested comparisons in TEWL made across ASSESSMENTS, PERIODS, and DAYS, as well as all possible interactions. No significant main effect differences of TEWL in ASSESSMENT data collection points and DAYS were identified. However, significant differences in TEWL were found across PERIODS (F (2, 18) = 17.49; p < 0.001). It should be noted that all mean scores are based on Least Squares Mean estimates. In examining the differences in TEWL across PERIODS, TEWL scores during the Pre-SSC period (LSMeans = 22.54) were significantly lower than TEWL scores during the SSC period (LSMeans = 28.98) (Tukey-Kramer adjusted p < 0.001). TEWL scores during Pre-SSC (LSMeans = 22.54) were significantly lower than TEWL scores during Post-SSC (LSMeans = 27.55) (Tukey-Kramer adjusted p < 0.001). No significant differences were found comparing TEWL scores during SSC and TEWL scores during Post-SSC. None of the four interactions was significant.

**Table 5:** TEWL Means and SDs Across Periods for Day 1 and Day 5 of the Study

	Pre-SSC Mean ± SD	SSC Mean ± SD	Post-SSC Mean ± SD
Day one	23.07 ± 6.5	29.04 ± 10.2	28.64 ± 10.3
Day five	22.00 ± 4.73	28.92 ± 8.23	26.46 ± 6.51

### Infection

Only one infant developed a hospital-acquired infection during hospitalization and that infection was clinically evident 7 days after SSC ended. None of the mothers reported any signs or symptoms of infection during the first thirty days post-discharge.

## Discussion

The study investigated the effects of 5 consecutive days of 90 minutes SSC sessions on stratum corneum hydration and transepidermal water loss by recording and comparing values on Days 1 and 5 while infants were in an incubator (30 minutes pretest), in SSC (90 minutes test), and back in an incubator (30 minute posttest). The number of infections during and after hospitalization were tracked and showed that only one infant had a positive blood culture seven days after SSC stopped and no infants had signs of infection within 4 weeks of discharge. Reviewing the risk factors for developing infection revealed that the infant was at higher risk of infection because he was on CPAP and had an intravenous catheter longer than other infants; these findings are consistent with Cohen-Wolkowiez and colleagues [34]. Because SSC's positive effect on reducing number of infections has been found to be limited to the time when SSC is being given or only shortly thereafter [35], repeated sessions of SSC need to be conducted to determine prevention of infection.

Stratum corneum hydration and transepidermal water loss are indications of skin barrier function in preterm infants. Stratum corneum hydration, while controlling for transepidermal water loss, increased from pre-SSC to SSC and decreased in post-SSC on Days 1 and 5 of the study. Thus, SSC contributed to better stratum corneum hydration and did so probably because skin-to-skin contact provides a degree of occlusivity at the skin surface, thereby also causing skin wetness. Skin wetness contributes to higher transepidermal water loss readings, which were present during SSC. The increased hydration finding is similar to the effects of skin occlusion in diapered areas in infants during the first 2 - 3 weeks of life but not at 4 weeks of life due to skin maturation [36, 37]. Transepidermal water loss is known to be increased in infant skin areas in which skin-to-skin contact occurs, such as in the cubital fossa when flexion of the forearm occurs due to newborn postures [38].

Additionally, residual effects of SSC were seen. Skin hydration was higher after SSC than before SSC on both days, and skin hydration was higher before SSC on Day 5 than it was on Day 1, suggesting that the increases in skin hydration were not just momentary, but lasting. Such a possibility is congruent with the physiology of the skin in that skin hydration changes are commonly detectable for several days [39, 40]. Residual effects of SSC have been found for 1 - 2 hours after SSC terminates [41], so these results contribute new information related to length of residual effects of SSC. Residual effects on TEWL also were suggested by data showing that TEWL after SSC was higher than TEWL before SSC, possibly due to SSC or maturation of the infant's skin over the five days of study [42].

The increase in stratum corneum hydration during SSC is an important finding. A hydrated stratum corneum maintains flexibility and is able to strengthen the cornified cell envelopes, increasing the strength of the corneocytes responsible for the strength of the stratum corneum [43]. Good moisturization (hydration) of the skin allows the stratum corneum to perform fully and optimizes the ability of the body to resist pathogens. Enhanced hydration also optimizes strength and flexibility of the skin, preventing an influx of unwanted chemicals and inflammatory responses [44]. Good stratum corneum hydration is the most basic need for adequate skin barrier function.

Two ways to hydrate the skin exist. One way is to conserve the skin's natural moisture by using an ointment; the other way is to add moisture. In SSC mother's chest, temperatures increase and sweating may occur [26, 45, 46], introducing maternally produced hydration into the skin-to-skin interface. Thus, SSC may work as an occlusive moisturizing agent that adds moisture and has an immediate hydration effect on the stratum corneum, as mentioned before. Yet, the amount of SSC producing any given amount of additional water at the skin surface is not known and needs to be investigated because excess hydration or prolonged hydration may predispose the skin to inflammation and irritation [47]. Fortunately, the stratum corneum usually gains only minimal moisture from the addition of water to the skin surface [44], suggesting that the 2-hour sessions of SSC may not be a problem, but the effects of repetitive SSC and/or 24/7 SSC on skin integrity need to be evaluated because prolonged occlusion may also affect skin microbial flora, hydration and TEWL [48].

The levels of transepidermal water loss recorded during the incubator periods were expected because TEWL remains high in preterm infants for several weeks after birth [49]. The increases in TEWL during SSC were probably due to the mother adding to the moisture available at the SSC interface that was available for evaporation rather than the infant's skin bringing more water to the surface that was available for evaporation. Accordingly, water came to the infant's outer layers of skin, raising the possibility that SSC may act as a mechanical humectants (an agent, usually chemical, that attracts the water from inner skin layers to the outer layer of the skin [50]), and further studies should be conducted to quantify maternal contributions to the high TEWL values. Additionally, Wesley and Malbach explained that, in addition to finding difference between black and white skin, TEWL of both races increases with skin temperature increase [51]. In the current study, infant skin temperatures increased during the two hours of SSC and the increase was similar to that previously noted in many other studies [52]. Because high TEWL implies concomitant loss of heat [23], one might think the thermal management of the preterm neonate during SSC could be compromised; however, body heat loss does not occur during SSC [52, 53] and basal metabolic rate does not rise when the infant's temperature rises [54] because mothers conduct heat to the infant during SSC [25, 26, 46], rather than the infant increasing heat production by his/her own metabolism.

The number of infection results need to be considered cautiously because of the small sample size and studies with larger sample size are warranted to determine the effects of SSC on infection rate. Nonetheless, SSC may help reduce the number of infections in other ways than modifying skin hydration. For example, SSC reduces infant stress [55, 56]. Stress affects the epidermis and the stratum corneum in ways that disrupt skin barrier functioning [57] by increasing corticosteroid secretion which reduces the production of lipids and intercellular lamellae in the stratum corneum, reducing skin barrier strength and water content of the skin [44, 58]. Endogenous glucocorticoids increase permeability of the skin barrier, contributing to stress-induced infections [59]. Thus, the multiple changes occurring in the skin of the stressed infant places the infant at risk for skin barrier dysfunction and infants in intensive care are usually stressed and have higher than normal levels of circulating cortisol [55, 60, 61]. SSC may minimize skin barrier dysfunction by ameliorating stress [22, 55, 56].

A limitation of the study was that statistical control for ambient humidity was not possible due to the small number of ambient humidity data points (ambient humidity was taken at the beginning of each period, not at each assessment). Because humidity influences transepidermal water loss, future studies should include more frequent measurement of ambient humidity. Future studies should also include equal numbers of dark- and light-pigmented skin subjects as well as measuring skin barrier function at different skin areas (i.e. the infant's back) during each period to control for site difference and occlusion effects. Also, future studies could examine maternal skin hydration and transepidermal water loss simultaneous to the infant's and test for any correlations between maternal and infant's values to determine presence of physiologic synchrony as a mechanism for change. Especially because physiologic synchrony between mothers and infants during SSC has been found in many biomarkers [26, 45, 46, 62-65].

### Conclusions

Hospital acquired infection is an important problem in preterm infants [1]. The data reported here are encouraging and suggest that SSC may be an occlusive agent to promote skin barrier functioning and assist the infant's underdeveloped immunoprotective functions in minimizing the possibility of hospital-acquired infections. The study has begun fulfilling the recommendation that SSC be tested as a strategy to improve skin hydration [4], but reveals that evaporative loss may be higher during SSC than during incubator care, and that the higher transepidermal evaporated water loss values may not necessarily be detrimental because few infections occurred even in its presence. Until definitive data are obtained through randomized controlled trials, clinical application of SSC to improve barrier functions and be recognized as a mechanism contributing to reduced infections is premature.
